# Using Interleaved Stimulation to Measure the Size and Selectivity of the Sustained Phase-Locked Neural Response to Cochlear Implant Stimulation

**DOI:** 10.1007/s10162-020-00783-y

**Published:** 2021-01-25

**Authors:** Robert P. Carlyon, François Guérit, John M. Deeks, Andrew Harland, Robin Gransier, Jan Wouters, Simone R. de Rijk, Manohar Bance

**Affiliations:** 1 Cambridge Hearing Group, MRC Cognition & Brain Sciences Unit, University of Cambridge, 15 Chaucer Rd, Cambridge, CB2 7EF England; 2 Dept. of Neurosciences, ExpORL, KU Leuven, Herestraat 49 box 721, 3000 Leuven, Belgium; 3 Cambridge Hearing Group, Dept. Clinical Neurosciences, University of Cambridge, Cambridge Biomedical Campus, Cambridge, CB2 0QQ England

**Keywords:** cochlear implants, neural nonlinearity, cortical response, psychophysics, electrophysiology

## Abstract

We measured the sustained neural response to electrical stimulation by a cochlear implant (CI). To do so, we interleaved two stimuli with frequencies F1 and F2 Hz and recorded a neural distortion response (NDR) at F2-F1 Hz. We show that, because any one time point contains only the F1 or F2 stimulus, the instantaneous nonlinearities typical of electrical artefact should not produce distortion at this frequency. However, if the stimulus is smoothed, such as by charge integration at the nerve membrane, subsequent (neural) nonlinearities can produce a component at F2-F1 Hz. We stimulated a single CI electrode with interleaved sinusoids or interleaved amplitude-modulated pulse trains such that F2 = 1.5F1, and found no evidence for an NDR when F2-F1 was between 90 and 120 Hz. However, interleaved amplitude-modulated pulse trains with F2-F1~40 Hz revealed a substantial NDR with a group delay of about 45 ms, consistent with a thalamic and/or cortical response. The NDR could be measured even from recording electrodes adjacent to the implant and at the highest pulse rates (> 4000 pps) used clinically. We then measured the selectivity of this sustained response by presenting F1 and F2 to different electrodes and at different between-electrode distances. This revealed a broad tuning that, we argue, reflects the overlap between the excitation elicited by the two electrodes. Our results also provide a glimpse of the neural nonlinearity in the auditory system, unaffected by the biomechanical cochlear nonlinearities that accompany acoustic stimulation. Several potential clinical applications of our findings are discussed.

**Supplementary Information:**

The online version contains supplementary material available at 10.1007/s10162-020-00783-y.

## Introduction

For many cochlear implant (CI) listeners, the pathway from implantation to speech comprehension is straightforward. The thresholds (Ts) and most comfortable levels (MCLs) for each electrode are obtained from the listener’s subjective response during the initial fitting session, additional adjustments are made during follow-up sessions, and speech perception gradually improves to the point where open-set sentence comprehension is possible, at least in quiet backgrounds (e.g. Firszt et al. 2004). However, infants, young children, and some adults cannot provide reliable subjective reports, and so objective measures of the neural response are required. Objective measures are also useful for diagnosing cases where a patient reports that sounds are inaudible or blurred. Finally, an objective measure of perception could aid the development of new treatments or stimulation methods that are initially tested in animal models, by allowing that evaluation to be performed using the same methods and stimuli as can be employed with humans. Examples of such novel methods include optogenetic and intra-neural stimulation (Middlebrooks and Snyder 2007; Dieter et al. 2019) and pharmaceutical interventions designed to improve the fidelity of the neural response (Chambers et al. 2017; Carlyon et al. 2018).

Ideally, an objective measure should be obtained using stimulation parameters that are very similar to those used clinically, and provide information on both the size and the selectivity of the neural response. Unfortunately, the large electrical artefacts that accompany CI stimulation have required researchers and clinicians to use stimuli that differ from those presented by the CI in everyday use. The most common solution has been to present electrical pulses at a very slow rate and to record the neural response in the gaps following each pulse. This solution is widely used in the measurement of the electrically evoked compound action potential (ECAP) and the electrically evoked auditory brain response (EABR). Unfortunately, the large difference between those pulse rates and the rates implemented in speech-processing strategies limits their usefulness. For example, the ECAP is typically measured using a pulse rate of 80 pps or below, and ECAP thresholds correlate only modestly with thresholds at the much higher pulse rates used in clinical speech-processing strategies (Cafarelli Dees et al. 2005; McKay et al. 2005; Potts et al. 2007). Previous studies have successfully measured the electrically evoked auditory steady-state response (EASSR) using amplitude-modulated (AM) pulse trains with moderately high carrier pulse rates of 500–900 pps, by processing the recorded EEG so as to remove the artefact from each pulse. However, the maximum pulse rate that can be used with this method is still limited, and recording remained restricted to electrodes contralateral to the CI (Hofmann and Wouters 2010; Gransier et al. 2016; Gransier et al. 2020).

**Fig. 1:**
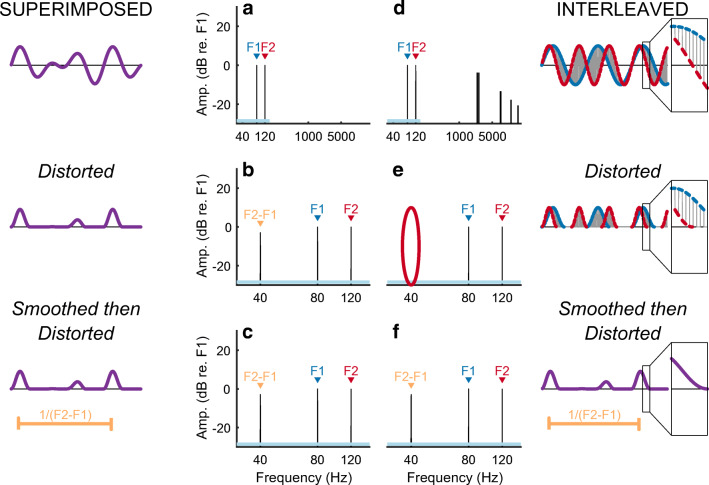
A) Frequency spectrum of an analogue dyad consisting of two superimposed sinusoids F1 (80 Hz) and F2 (120 Hz). A 35-ms portion of the waveform is shown on the left. B) An instantaneous nonlinearity (such as could occur in the stimulating device), consisting of half-wave rectification and squaring, results in a DP with frequency F2-F1 = 40 Hz. This DP is also observed when the nonlinearity is preceded by smoothing, such as may occur in the auditory system (C). D) Stimulus produced by interleaving 200-μs sections of 80- and 120-Hz sinusoids. A zoomed-in section of the waveform is shown in the box on the far right. The nonlinearity now fails to produce a DP (part E, red oval) unless preceded by smoothing (F)

**Fig. 2:**
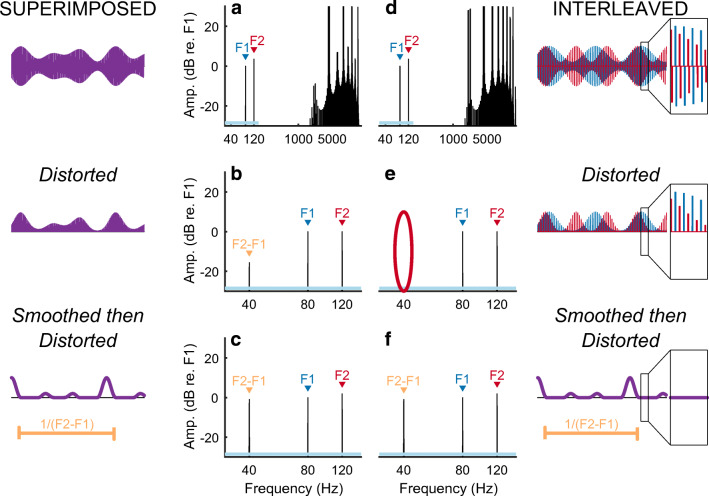
Format is similar to Fig. 1. A) Amplitude-modulating a 4644-pps pulse train by the sum of two modulators (F1 + F2 Hz) produces a DP when the stimulus is passed through a nonlinearity (B). A somewhat larger DP arises when the nonlinearity is preceded by temporal smoothing (C). Interleaving two 2322-pps pulse trains that are separately modulated at F1 and F2 Hz (part D) does not produce a DP when the stimulus is passed through a nonlinearity (red oval, part E). Preceding the nonlinearity with temporal smoothing causes the DP to re-appear (part F)

Here we use a method that is qualitatively different from those employed previously and that does not rely on the identification and subsequent removal of the electrical artefact. The method can be used with extremely high (> 4000 pps) pulse rates or, in principle, with quasi-analogue stimuli, and allows one to record from any electrode on the scalp including those adjacent to the implant. We use the method to characterize both the nonlinearity and the selectivity (spread-of-excitation) of the sustained auditory neural response to CI stimulation. Our approach is based on two assumptions. The first is that the neural response to electrical stimulation is nonlinear. One well-known neural nonlinearity is that electrical stimuli (such as sinusoids or biphasic pulses) containing equal-sized positive and negative deflections can elicit action potentials, which are always highly asymmetric. It is also known that the function relating firing rate to input current is not linear over a neuron’s entire dynamic range (van den Honert and Stypulkowski 1987; Miller et al. 1999). Therefore, if we superimpose two stimuli of frequencies F1 and F2 Hz on a single electrode, we may observe a “neural distortion response (NDR)” at a frequency related to both F1 and F2 Hz, such as the difference frequency F2-F1 Hz. Parts A and B of Figure 1 illustrate this for the case of sinusoidal stimulation followed by two nonlinearities, namely half-wave rectification and squaring. Unfortunately, the CI device may also be nonlinear, and so the presence of a component at F2-F1 Hz would not necessarily reflect a neural response. The second assumption is that neural nonlinearities are not applied directly to the instantaneous electrical stimulus but are preceded by biological temporal dependencies that include smoothing, such as occurs initially when the electrical charge is integrated by the neural membrane prior to the production of action potentials. Additional temporal dependencies manifest in the phenomena of accommodation, facilitation, refractoriness, and adaptation (Boulet et al. 2016). Our ALFIES (Alternating-Frequency Interleaved Electrical Stimulation) method interleaves stimulation of stimuli with frequencies of F1 and F2 Hz, or pulse trains that are amplitude modulated at F1 and F2 Hz. The reason for the interleaving is that instantaneous nonlinearities, such as those produced by stimulating devices, should not produce distortion products (DPs) at frequencies, such as F2-F1 Hz, that depend on both F1 and F2. This is illustrated in Fig. 1D, where 200-μs steps from two sinusoids are interleaved so that any one time-point now contains information about only one sinusoid. Segmenting the sinusoids produces some additional high-frequency components in the spectrum, but interleaving them means that DPs are absent even after an extreme nonlinearity, namely rectification followed by squaring (Fig. 1E). Importantly, when the stimulus is smoothed prior to the nonlinearity, a component at F2-F1 Hz does appear (Fig. 1F). This F2-F1-Hz component can arise from a neural nonlinearity that is preceded by smoothing, but not from an instantaneous nonlinearity inherent to the stimulating device. Figure 2 illustrates the same process for interleaved pulse trains that are amplitude modulated (AM) at F1 and F2 Hz respectively. These simulations are described in detail in the “Methods” section, where we also demonstrate mathematically that the reasoning applies to all instantaneous nonlinearities, and not just the rectification and squaring used in Figs. 1 and 2. The effect of smoothing can be thought of as “undoing” the interleaving, so that each time point of the combined stimulus depends both on the F1-Hz and F2-Hz waveforms, thereby allowing subsequent nonlinearities to produce a component that depends on both F1 and F2. We stress that the smoothing must occur *before* the nonlinearity in order for a component at F2-F1 Hz to appear.

The experiments described below include measurements obtained from stimulation of a single electrode using interleaved analogue dyads (cf. Fig. 1) and AM pulse trains (Fig. 2) for the case where F2-F1 has a frequency between about 90 and 120 Hz, typical of neural responses generated by the upper brainstem (Herdman et al. 2002; Bidelman 2015). Calibration measures obtained with fresh-frozen cadaver heads showed that interleaving F1- and F2-Hz tones substantially reduced, but did not eliminate, the artefactual distortion product produced by superimposed sinusoids (Figs. 1A–C). Measures with living CI users of the Advanced Bionics device found only this artefactual response and did not reveal any neural response. We then obtained measurements using interleaved AM pulse trains from living users of the CI manufactured by Cochlear and showed that, consistent with the results for analogue dyads, a neural response could not be obtained for F0s (= F2-F1) of 90 or 120 Hz. However, the use of AM pulse trains allowed us to use lower F0s without extending the duration over which the stimulus is charge-imbalanced, and to observe a genuine neural distortion response (NDR) for F0s close to 40 Hz. We show that the NDR has a group delay of 45 ms, typical of a source in auditory cortex and/or thalamus, and use this response to measure the spread of excitation in the sustained neural response to stimulation of CI electrodes. We then compare the results to EASSRs using methods developed for the same device and conclude with a description of the potential scientific and clinical applications of our results.

## Methods

### Simulations

#### Analogue Dyads (Fig. 1)

The basic stimulus consisted of two simultaneous 1-s sinusoids having frequencies of F1 = 80 Hz and F2 = 120 Hz, generated in MATLAB. To generate the interleaved stimulus, each sinusoid was partitioned into 200-μs contiguous segments, and the odd-numbered segments of F1 were interleaved with the even-numbered segments of F2. Power spectra were calculated using the MATLAB *fft* function and are shown, alongside a portion (slightly more than one period) of the waveform, for the standard (“superimposed”) and interleaved stimuli in Fig. 1 A and D respectively. Distortion (Fig. 1B, E) consisted of half-wave rectification followed by squaring. Smoothing (Fig. 1C, F) was implemented using a 30th-order low-pass IIR filter having a half-power frequency of 300 Hz, using the MATLAB function *designfilt* with the parameter “*lowpassiir*”.

#### Pulse Trains (Fig. 2)

Stimuli consisted of two trains of negative-first symmetric biphasic pulses, with each phase of each pulse having a duration of 25 μs and with a zero inter-phase gap. One pulse train was sinusoidally amplitude modulated (AM) at 80 Hz (F1) and the other was modulated at 120 Hz (F2); the modulation depth was 100 % in both cases. For the interleaved stimulus (Fig. 2D) the pulse rate for each train was 2322 pps and the two pulses trains were interleaved such that each pulse from F1 fell exactly mid-way between two pulses from F2, and vice versa*.* This resulted in a composite pulse rate of 4644 pps. For the superimposed stimulus (Fig. 2A) we amplitude-modulated a 4644-pps pulse train by the sum of 80- and 120-Hz modulators, both having a 100 % modulation depth, and then divided by 2. Smoothing and distortion (Figs. 2B, C, E, F) were the same as for the analogue dyads of Fig. 1.

All simulations described above were generated using a sample rate of 1 MHz. The Matlab code is publicly available online (Guérit and Carlyon 2020). We note that instantaneous nonlinearities do not produce a component that is absent from the spectrum of either F1 or F2 when each is passed through that nonlinearity separately, and that this is true for any instantaneous nonlinearity and not just the rectification and squaring illustrated here. To illustrate this, consider an interleaved stimulus *x*_*int*_ such that *x*_*int*_(*t*) = *x*_1_(*t*) + *x*_2_(*t*) at any time *t*, and that at any time either *x*_1_(*t*) or is *x*_2_(*t*) set to zero. Therefore for any instantaneous nonlinearity *g* and at any time t, *g*(*x*_*int*_(*t*)) is always equal to either *g*(*x*_1_(*t*)) or *g*(*x*_2_(*t*)). In other words, at any time *t*, *g*(*x*_*int*_(*t*)) = *g*(*x*_1_(*t*)) + *g*(*x*_2_(*t*)). Because the Fourier Transform is a linear operation (Smith 1997), it follows that at any frequency f and with $\hat{g}$ being the Fourier transform of *g*, $\hat{g}\left({x}_{int}(f)\right)=\hat{g}\left({x}_1(f)\right)+\hat{g}\left({x}_2(f)\right)$. Hence the spectral content of the distorted, interleaved stimulus is a simple sum of the real and imaginary parts of the spectra of the distorted *x*_1_ and of the distorted *x*_2_. Because neither of these two spectra contain a component at any frequency that depends both on *x*_1_ and on *x*_2_, there will therefore be no energy at these frequencies in the spectrum of the distorted interleaved stimulus. Importantly, if smoothing is applied to the interleaved stimulus then *g*(*x*_*int*_(*t*)) is no longer always equal to either *g*(*x*_1_(*t*)) or *g*(*x*_2_(*t*)) and so components that depend on both *x*_1_ and on *x*_2_ may appear in the spectrum.

### EEG Experiments

The EEG experiments used two types of stimuli, namely analogue dyads (cf. Fig. 1) and interleaved AM pulse trains (cf. Fig. 2). Approval for the study was granted by the National Research Ethics Committee for the East of England. A copy of the informed consent form that was signed by the participating CI listeners is provided in the Appendix.

### Analogue Dyads

The analogue dyads shown in Fig. 1 were approximated using an Advanced Bionics CI controlled by the BEDCS research software and hardware provided by the manufacturer. The step size was 126.26 μs and each period (F0 = F2-F1 Hz) consisted of 72 steps, leading to an F0 of 110.0023 Hz. For brevity, we will refer to this F0 as 110 Hz and to the two component sinusoids as F1 = 220 Hz and F2 = 330 Hz. The amplitude was constant during each step (unlike Fig. 1D), leading to some additional high-frequency components in the spectrum related to the 126.26-μs step size. The amplitude resolution was 8 bits. Each stimulus had a duration of 300 ms, equal to exactly 33 periods of the F0. All stimuli were presented in monopolar mode.

Five users of the CI manufactured by Advanced Bionics took part; their details are given in the first five rows of Table 1. Prior to the start of the recordings, the Ts and MCLs were obtained separately for three different stimuli by initially presenting each stimulus at a sub-threshold level, and asking the subject to indicate the presence and loudness of the stimulus using an 11-point chart. The level was then gradually increased until the subject indicated that it was audible (threshold, level 1) and then until it reached MCL (level 6). The three stimuli consisted of the interleaved dyads with F1 and F2 both presented to electrode *x*, both presented to electrode *y*, or with F1 presented to *x* and F2 presented to *y*. Electrodes *x* and y were numbers 8 and 9 for all listeners except AB31 for whom electrodes 3 and 4 were used. The lowest MCL from these three measures was then used for all EEG recordings (in all three conditions) for that listener. Each condition consisted of 1000 repetitions of the 300-ms stimulus, each separated by a silent gap of approximately 600 ms, except for the condition where F1 and F2 were interleaved on adjacent electrodes, in which case 2000 repetitions were obtained. Each presentation was accompanied by a trigger that was routed to the recording system via a custom-built triggering interface,^1^ so that the traces could be averaged.

**Table 1:** Details of the experimental subjects. The prefix “AB” refers to subjects implanted with an Advanced Bionics device and the prefix “C” indicates a Cochlear device. The device type refers to the specific model of the internal part of the CI produced by the manufacturer. For Advanced Bionics implants the HiFocus1J is a lateral wall array whereas the HiFicus ms is mid-scalar. For the Cochlear device, the abbreviation “CA” refers to the Contour Advance (perimodiolar) electrode array. “PM” refers to a curved perimodiolar electrode array. The CI512 device also uses a perimodiolar array. All other Cochlear devices used a straight array

Subject code	Ear tested	Device type	Array	Age	CI use (months)
AB01	L	HiRes90k	HiFocus 1J	73	113
AB02	L	HiRes90k	HiFocus 1J	59	130
AB26	L	HiRes90k	HiFocus ms	57	51
AB31	R	HiRes90k	HiFocus 1J	39	195
AB32	L	HiRes90k	HiFocus 1J	67	117
C09	R	CI24RE CA	Curved	69	155
C12	L	CI522	Straight	67	45
C18	L	CI24RE CA	Curved	66	53
C19L	L	CI512	Curved	66	29
C19R	R	CI422	Straight	66	52
C27	R	CI512	Curved	70	59
C29	L	CI522	Straight	76	28
C30	R	CI512	Curved	71	34

To assess whether any nonlinearities inherent to the Advanced Bionics CI or to the recording device could result in a distortion product, we also obtained similar recordings using a HiFocus 1J lateral wall electrode array implanted in a fresh-frozen cadaver head, after the cochlea had been flushed with 1.0 % saline through the lateral semi-circular canal. The surgery was performed using methods described in de Rijk et al. (2020). The dyads were presented in an interleaved or superimposed manner to electrode 3, and also in an interleaved manner with F1 presented to electrode 3 and F2 presented to electrode 4. The peak current level was 100 μA, compared with a mean value of 33uA (range 22–39 μA) used for the five living AB subjects tested; this was done so as to increase the level of any distortion relative to the noise floor, thereby making it easier to measure. A total of 300 dyads, each with a duration of 0.99998 s (equal to 110 periods) were presented with a gap of approximately 200 ms between presentations, and with a trigger sent to the EEG system at the start of each dyad. Recording electrodes were presented at locations close to P9/P10 (ipsilateral to the implant) and the chin in the 10/20 system and referenced to an electrode close to Cz. Recording electrode positions for the living participants are described below.

### AM Pulse Trains

Eight adult patients implanted with a CI manufactured by Cochlear Ltd. took part. All were post-lingually deafened and used their CI in everyday life. One subject’s data were characterized by a high noise level and are not presented or analysed here. Subject C19 was bilaterally implanted and we recorded EEG traces to stimuli presented to both ears separately, leading to a total of eight ears being tested (bottom eight rows of Table 1).

Each stimulus consisted of the sum of two *y*-pps trains of 25 μs/phase cathodic-leading symmetric biphasic pulses. The two pulse trains were interleaved so that each pulse of one train fell exactly mid-way between two pulses of the other train, leading to a composite rate of 2*y* pps. The value of *y* varied somewhat between conditions but was typically 2322 pps (see below), in which case the composite rate was 4644 pps (cf. Fig. 2). Each pulse had an inter-phase gap of 8 μs. Each pulse train was amplitude modulated (AM), with modulation rates of F1 and F2 Hz, where F2 = 1.5F. The AM was sinusoidal in linear current between a comfortably loud and a threshold level, as described below (Gransier et al. 2016). Stimuli were generated using research software (Nucleus Implant Communicator 4, “NIC4”) provided by Cochlear Ltd., and controlled using Matlab running on a battery-powered laptop. The laptop was connected via an optically isolated interface box (POD, Cochlear Ltd.) to a coil similar to that used clinically, placed on the scalp and over the subject’s implanted receiver-stimulator. The two pulse trains were presented to the same apical electrode (usually electrode 20) for all experiments except for the tuning experiment, where they could be presented to the same or different electrodes. All stimuli consisted of 300 1-s epochs played continuously, with a trigger pulse sent to the EEG system, via the custom trigger interface at the start of the epoch in order to aid analysis. The presentation of each condition therefore took 5 min. All stimuli were presented in monopolar (“MP1 + 2”) mode, as in clinical use.

Prior to the start of the experiment, and for each electrode that was stimulated, we estimated the threshold and most comfortable loudness (MCL) level for an unmodulated 1-s. 4644-pps pulse train. This was done by presenting the appropriate stimulus initially at a sub-threshold level and asking the subject to indicate the loudness (or presence) of the stimulus using an 11-point chart. The current was then gradually increased until the subject indicated that it was audible (threshold, level 1) and then until it reached MCL (level 6). These thresholds and MCLs were noted and used as the minimum and maximum values for the modulated stimuli to be used in the EEG recordings.

The recordings started with the measurement of the amplitude and phase of the response for pulse trains interleaved on a single electrode and with F0 (= F2-F1) equal to 37, 40, and 43 Hz. We used three closely-spaced modulation frequencies in order to measure the group delay of the response, which can be derived from the slope of the function relating the phase of the response to the modulation frequency. There were 54 pulses per period of F0 such that the pulse rate *y* for each pulse train was 1988, 2160, and 2322 pps respectively. The pulse trains were presented to electrode 20 (“e20”) for all subjects except C09 for whom e19 was used because e20 was disabled in her standard clinical map. Her data were plotted and analysed as if we had stimulated e20. For five subjects (C18, C19L, C19R, C27, and C29) we next obtained the same measures at AM rates of 37 and 40 Hz with the pulse rate set to 2322 pps (same as at 43 Hz, leading to a non-integer number of pulses per period of F0 at 37 and 40 Hz) and obtained very similar results, which are not presented here, except to provide an estimate of test-retest reliability. A “tuning” experiment was then performed using a single AM rate of 43 Hz. Here the interleaved pulse trains were presented either to the same electrode, which was 1, 6, or 12 electrodes basal to the one used for the first experiment, or with F2 presented to one of those more basal electrodes and with F1 presented to the most apical electrode.

To study the effect of the overall modulation rate used, the first set of pulse train measurements was repeated for subjects/ears C18, C19L, and C19R using F0s of 87, 90, and 93 Hz and of 117, 120, and 123 Hz. The spatial selectivity measures for subject C30 were tested in a separate session that took place after the other measurements for that subject. His MCLs were measured afresh for that second session and, for electrode 20 (which was tested in both sessions), had dropped by 8 clinical current units (slightly more than 1 dB).

We additionally measured the EASSR in seven ears for AM rates of 37, 40, and 43 Hz, and for 500-pps pulse trains, using an analysis method identical to that described by Gransier et al. (2016, 2020), and that involved identifying and blanking the stimulus pulses in the recording and linearly interpolating across the blanked values. The pulse trains were amplitude-modulated in linear current between the T and MCL for an unmodulated 500-pps pulse train. This condition differed from that described by Gransier et al. (2016) primarily in the use of the hyper-rate BioSemi system (see below), which allowed more accurate sampling of the electrical artefact corresponding to each pulse. As in that study and in Gransier et al. (2020), post-processing was performed so as to blank the electrical artefact for each pulse and to linearly interpolate across this blanked period before performing the FFT. In all the analyses presented here, the blanked periods extended from 0.2 ms before to 1.4 ms after each pulse (cf. Gransier et al. 2016; Gransier et al. 2020).

### EEG Recording System and Analysis

EEG recordings were obtained using an 8-channel 24-bit system designed and built by the BioSemi company (Amsterdam, The Netherlands) to our specifications. This “hyper-rate” system is based on their standard systems but with a sampling rate of 262,144 kHz. The very high sampling rate is necessary for the accurate measurement of the electrical stimulation artefacts, which is important when using linear interpolation for removing the stimulation artefacts, as used in the measurement of EASSRs (Gransier et al. 2020), but which is not essential for the ALFIES method. In the BioSemi system, EEG signals are pre-amplified by Ag/AgCI active electrodes placed on the subject’s head. A cap was used to position the recording electrodes according to the international standardized 10/20 system. We placed electrodes at Fz, Fpz, Cz, Iz, P9, P10, and at the left and right mastoids. Except where otherwise stated, analyses are based on the average of the two mastoids and Iz, relative to Cz. Participants sat comfortably in a sound-treated and electromagnetically shielded booth and watched subtitled movies.

EEG recordings were cut into epochs, each starting with a trigger output from the interface with the implant. Because the internal clock of the CI differs from that of the BioSemi system, epochs were further re-cut to have an exact number of electrical pulses (or periods for the analogue dyads) per second. This avoided spectral splatter of the primaries in the subsequent FFT analyses. The epochs were finally averaged into a single 300-ms or 1-s epoch for the analogue and pulse-train stimuli, respectively. The amplitudes and phases at F0, F1, and F2 Hz were then obtained from the corresponding bins of an FFT of that averaged epoch. The power at F0 Hz was compared with that of the adjacent 12 bins (6 each side) using an *F* test (Dobie and Wilson, 1996). An F ratio greater than 10.92 (*p* < 0.01, approx. 6 dB signal-to-noise ratio) was deemed significant.

## Results

### Analogue Dyads

**Fig. 3:**
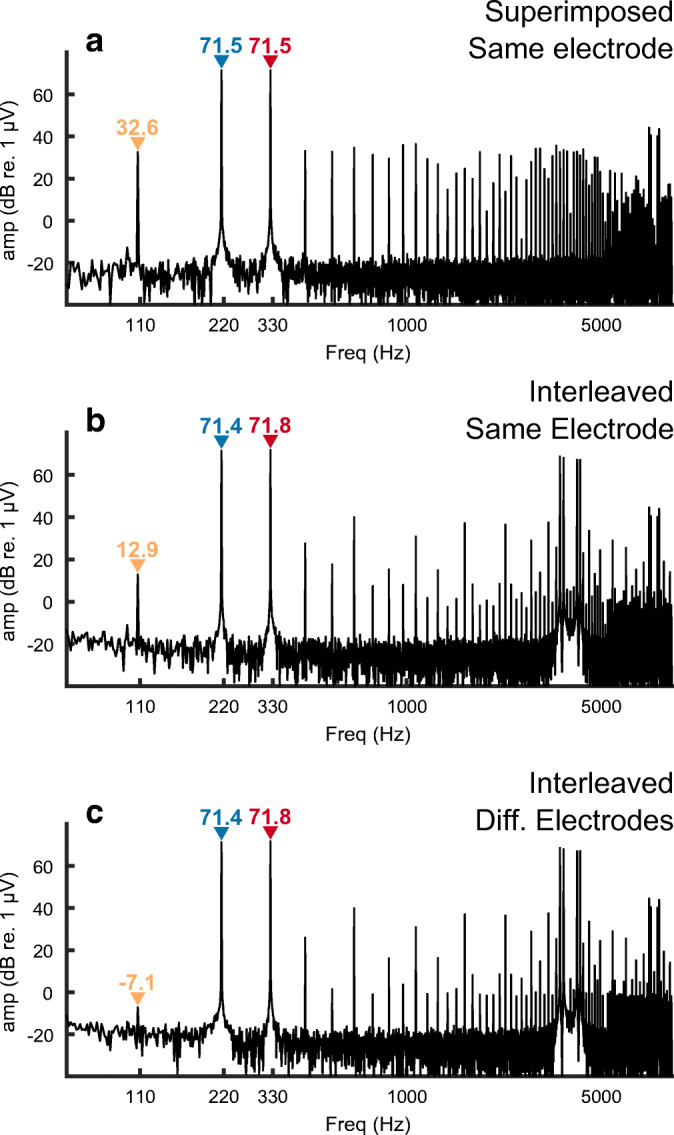
FFT of recordings from a fresh-frozen cadaver head in response to analogue dyads consisting of component frequencies of approximately F1 = 110 Hz and F2 = 220 Hz. Amplitudes of these primary frequencies are shown in each panel in blue and red text for F1 and F2, respectively. A distortion product at F2-F1 = 110 Hz is observed with an amplitude, indicated in orange text, that differs between (A) F1 and F2 superimposed, presented to the same electrode, B) F1 and F2 interleaved, presented to the same electrodes, and C) F1 and F2 interleaved and presented separately to adjacent electrodes

Figure 3 shows the results from the cadaver recordings in response to the analogue dyads presented via the Advanced Bionics device. Parts A and B show that interleaving the dyads on the same electrode reduced the artefact DP by 20 dB, compared with superimposed stimulation, and that the artefact level was about 58 dB lower than that of the primary components (F1 and F2). This shows both that interleaving the stimuli did substantially reduce the distortion and that, contrary to our simulations, a small residual artefact remained. Part C showed that the artefact was reduced by a further 20 dB when F1 and F2 were presented to adjacent electrodes, suggesting that it arose from the stimulating device rather than the EEG system. One possible reason comes from the fact that the circuitry of the Advanced Bionics CI contains capacitors that are present to ensure that stimulation remains charge-balanced. This would have partially smoothed the stimulus so that any subsequent nonlinearity could then produce an (attenuated) distortion product at F2-F1 Hz, even for interleaved stimuli. The device contains separate circuitry for each electrode, which could explain why the distortion product dropped even further when F1 and F2 were applied to different electrodes. Alternatively, some capacitance could arise from electro-chemical interactions between each electrode and the intracochlear saline.

**Fig. 4:**
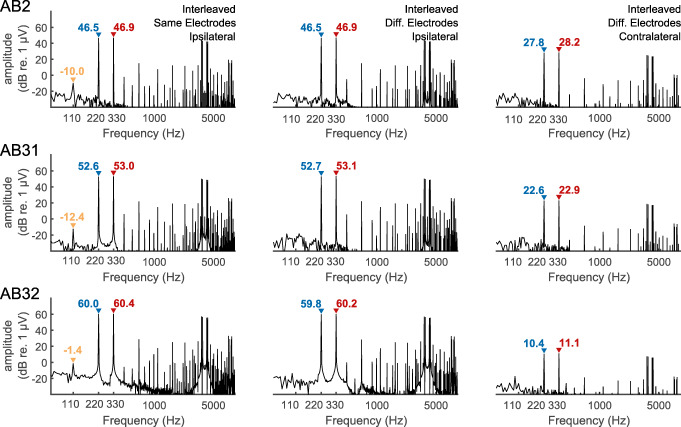
FFT of recordings from three subjects implanted with the Advanced Bionics device in response to analogue dyads consisting of component frequencies of approximately F1 = 110 Hz and F2 = 220 Hz. Amplitudes of these primary frequencies are shown in each panel in blue and red text for F1 and F2, respectively. A distortion product at F2-F1 = 110 Hz is observed with an amplitude, indicated in orange text when F1 and F2 are interleaved on the same electrode (left panels) but not when presented separately to different electrodes (right panels). The amplitude of the component at F2-F1 Hz is shown only when significantly greater than the background noise

The EEG recordings from the (living) CI listeners closely followed those observed from the cadaver, with a component at F2-F1 Hz observed when the interleaved stimuli were presented to the same, but not to different, electrodes. This is illustrated for three example listeners in Fig. 4, with the first two columns comparing the results for same- and different-electrode stimulation, and with recording from an electrode ipsilateral to the implant in each case. The third column shows results for different-electrode stimulation and with the recording electrode contralateral to the implant and illustrates the attenuation of the artefact relative to the case with ipsilateral recording (second column).

The results from the analogue dyads show that, for the stimuli used here, any truly neural distortion response is likely to be smaller than our noise floor of about − 25 dB/Hz re 1 μA at 110 Hz. They show that the ALFIES method can reduce artefactual distortion in a real CI, but highlight the need for additional checks to confirm that any distortion product is truly of neural origin. Fortunately, as described below for the AM pulse trains, those checks are quick and simple to perform.

### AM Pulse Trains Interleaved on the Same Electrode

**Fig. 5:**
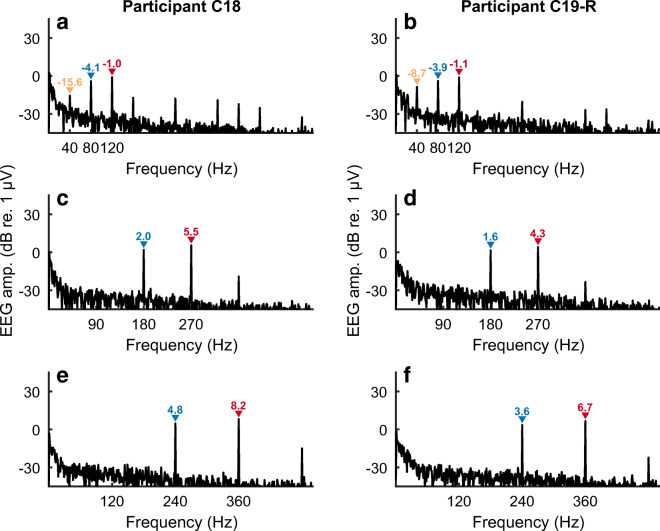
FFT of recordings from two subjects implanted with a cochlear device in response to interleaved pulse trains. The difference frequency (F2-F1 Hz) is either 40 Hz (panels A, B), 90 Hz (C, D), or 120 Hz (E, F). The amplitude of the component at F2-F1 Hz is shown only when significantly greater than the background noise

The responses obtained from Cochlear participants C18 and C19R in response to AM pulse trains when the F0 (F2-F1) is equal to 90 Hz are shown in Fig. 5 C and D, with corresponding data for an F0 of 120 Hz shown in Fig. 5 E and F, respectively. This extends the finding of no NDR for F0s in the range of about 90–120 Hz, obtained with Advanced Bionics participants and with analogue dyads, to AM pulse trains presented with the Cochlear device and to different subjects. Measures obtained with two Advanced Bionics participants, and not shown here, also failed to reveal an NDR for SAM pulse trains and with an F0 of 96 Hz. Hence there appears to be no fundamental difference between the responses obtained with the two makes of CI. We chose to focus on users of the Cochlear device because we wished to compare our results to EASSR measures obtained with that device, and for which we have developed sophisticated measures of the EASSR (Gransier et al. 2016; Gransier et al. 2020).

**Fig. 6:**
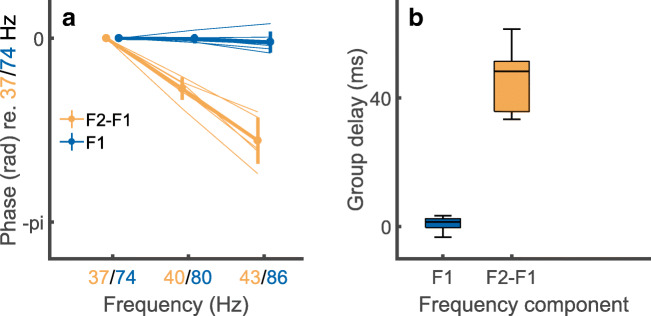
A) Phase vs frequency plots for the NDR (F2-F1 Hz, orange) and to an electrical artefact (F1 Hz, blue) in response to interleaved pulse trains having AM rates where F2-F1 is 37, 40, or 43 Hz. The abscissa shows F2-F1 Hz in orange and F1 Hz in blue. Solid lines show average data and faint lines show individual data. B) Box-and-whisker plots showing the group delay, derived from the functions shown in part A, for the NDR (orange) and F1-artefact (blue)

Figure 5 A and B show the results obtained with participants C18 and C19R, with the F0 of the SAM pulse trains equal to 43 Hz. In contrast to the results obtained at higher F0s (Figs. 5C–F), there is a clear component at 43 Hz. In the following, we present multiple checks to confirm this is a true neural distortion product. First, the variation in the phase of the component at F2-F1 Hz decreased monotonically from 37 to 43 Hz, for all listeners, consistent with a mean group delay of 45 ms, as shown by the orange lines and orange bar in Fig. 6 A and B respectively. This value is typical of a cortical and/or thalamic response, as obtained with both acoustic and electrical stimuli (Gransier et al. 2016; Gransier et al. 2017). The group delay at the primary stimulation frequencies (F1 and F2 Hz) was zero, as expected from an electrical artefact, and is shown for F1 by the blue lines and blue bar in Fig. 6 A and B. To assess test-retest reliability we compared the amplitude of the NDR at 40 Hz, obtained in the main measurements and with a carrier pulse rate of 2160 pps, to that obtained for five subjects in the additional measurements described in the “Methods” section at a carrier rate of 2322 pps. The mean values of − 14.1 and − 13.5 dB re 1 μV did not differ significantly (*t*(4) = 0.56, *p* = 0.6), and the standard deviation of the differences between the two measures for each subject was 2.2 dB.

**Fig. 7:**
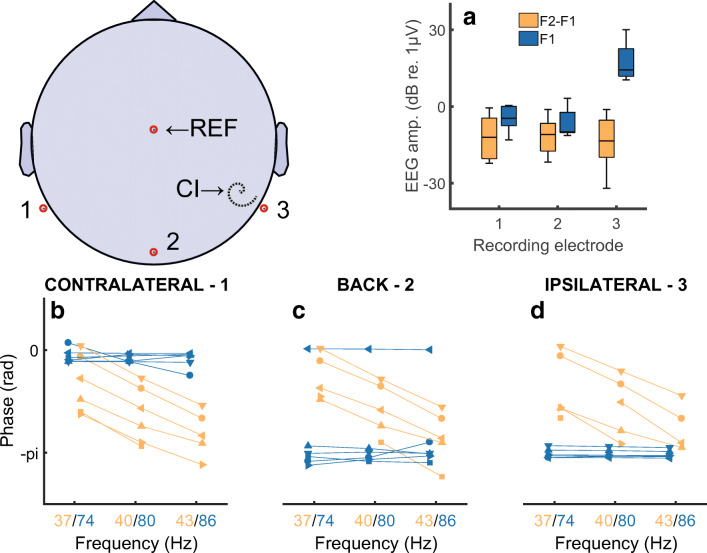
Part A) shows the amplitude of the response at the NDR for the NDR (F2-F1 Hz, orange) and to an electrical artefact (F1 Hz, blue) in response to interleaved pulse trains having AM rates such that F2-F1 Hz is close to 40 Hz. Responses are shown for three electrodes illustrated schematically on the left. Electrode 2 was Iz. Electrodes 1 and 3 were P9 and P10 or vice versa, depending on which ear was implanted. Parts B, C, and D show phase vs frequency plots for recording electrodes 1, 2, and 3, respectively. Each of these three plots shows the NDR (F2-F1 Hz, orange) and to an electrical artefact (F1 Hz, blue) in response to interleaved pulse trains having AM rates where F2-F1 is 37, 40, or 43 Hz

Further evidence that the component at F2-F1~40 Hz is a true NDR comes from the distribution of the response amplitude and phase responses across the different recording electrodes. Figure 7 A shows the amplitudes of the NDR at F2-F1 Hz and the artefact component at F1 Hz, measured at three different recording electrodes, namely P9, P10, and Iz. We refer to these electrodes as the ipsilateral, contralateral, and back electrodes, because the ipsilateral electrode could be either P9 or P10 depending on which ear was implanted. The amplitude of F1 Hz (blue bars) is, unsurprisingly, much greater for the ipsilateral recording electrode than for the other recording electrodes. In contrast, the amplitude of the NDR at F2-F1 Hz (orange bars) is roughly similar for all recording electrodes. Figure 7 B–D show that the slope of the function relating the response phase to F2-F1 Hz, and therefore the group delay, is similar at all recording electrodes. Hence one can obtain an uncorrupted measure of both the amplitude and phase of the sustained neural response, even when the recording electrode is immediately adjacent to the CI. This is because, for the interleaved stimuli used here, there is only a very small electrical artefact at F2-F1 Hz. An estimate of the maximum size of this artefact can be obtained from the noise floor of − 30 to − 33 dB re 1 μV, obtained with F2-F1 = 90 or 120 Hz (Figs. 5C–F), where no component at F2-F1 Hz was observed, if we assume that the electrical artefact is the same at all F0s. Because MCLs and hence the stimulus levels used were the same for all modulation rates tested, this is likely to have been the case. A novel feature of the ALFIES method is that it minimizes any artefact at F2-F1 Hz, thereby revealing an NDR at that frequency, rather than requiring one to remove the artefact by subsequently processing the recordings.

Previous measures of the EASSR have also observed larger and more consistent responses for modulation rates near 40 Hz, where phase-locked responses are likely to arise from the thalamus and auditory cortex (Herdman et al. 2002; Farahani et al. 2017; Luke et al. 2017), compared with frequencies between 90 and 120 Hz (Gransier et al. 2016). Although phase-locked responses at these higher frequencies can arise from a variety of sources (Coffey et al. 2019), they are likely to have a strong brainstem component (Herdman et al. 2002; Bidelman 2015). It is likely that the absent NDRs at 90–120 Hz are due, at least in part, to the same reasons why EASSRs are smaller and less reliable than at frequencies of about 40 Hz; these may include a greater distance from the brainstem generators to the scalp, and, possibly, CI users’ poorer phase locking at these higher rates at the level of the brainstem. Note however that CI users show good phase locking at the level of the auditory nerve, even at pulse rates that are too high to elicit an accurate temporal pitch (Carlyon and Deeks 2015). The potential origins of the nonlinearities and smoothing responsible for our observed NDRs are considered further in the Discussion section.

### Interleaved Pulse Trains Presented on Different Electrodes

**Fig. 8:**
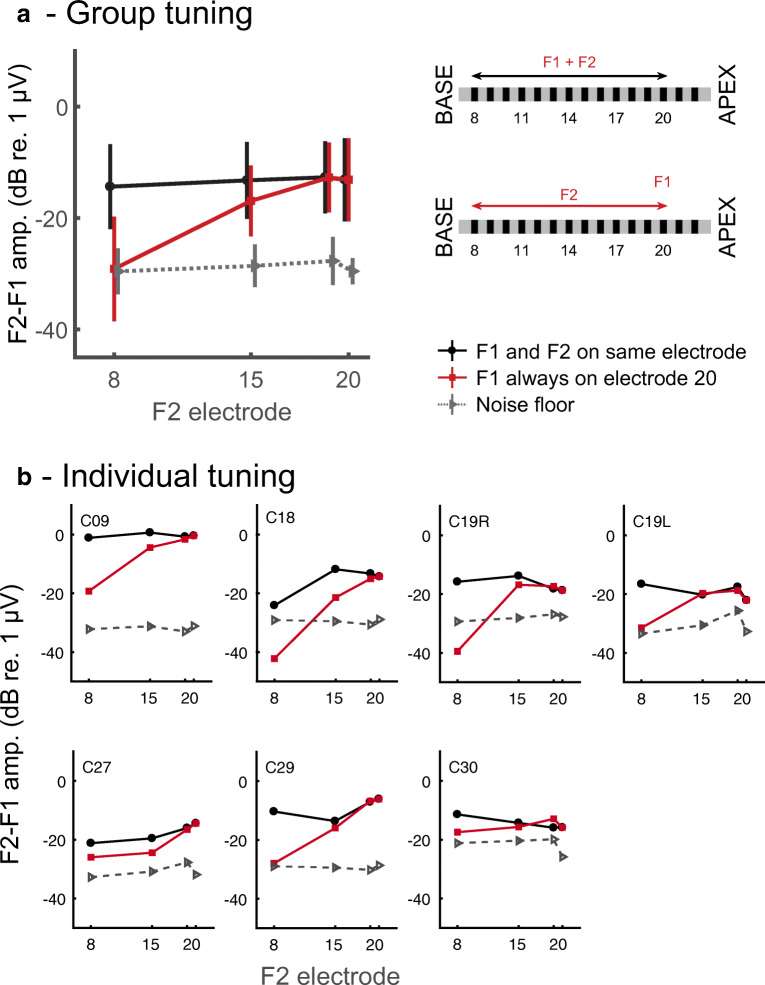
A) Average data and schematic of conditions in the spatial selectivity (“tuning”) experiment. The data plot shows the amplitude of the NDR as a function of the electrode that conveys the F2 stimulus. Red lines show the NDR amplitude when F1 is always presented to the most apical electrode tested (usually e20). Black lines show the NDR amplitude when F1 is presented to the same electrode as F2. Grey lines show the noise floor. B) Individual data

An assumption underlying the ALFIES method is that the NDR arises from the response of a common neural population that responds to both the F1-Hz and the F2-Hz waveforms that formed the composite stimulus. We therefore estimated the spatial selectivity of CI stimulation by presenting F1 and F2 on the same or on different electrodes and, in the latter case, measured the NDR amplitude as a function of the separation between those two stimulating electrodes. Figure 8 A illustrates the paradigm that we used along with the results averaged across listeners. When F1 and F2 were presented to the same electrode (black line and symbols) the NDR amplitude was, on average, about −16.5 dB re 1 μV regardless of whether that electrode was number 20, 19, 14, or 8. However, when F1 was always presented to electrode 20, near the apical end of the array for this device, the NDR decreased slightly when F2 was presented on electrode 14 and decreased substantially and into the noise floor when it was presented on electrode 8 (red line and symbols). These separations of 6 and 12 electrodes between the F1- and F2-pulse trains correspond to distances of approximately 4.5 and 9 mm for the Cochlear device used here. A two-way repeated-measures ANOVA revealed significant main effects of the factors “F2 electrode” (F(2,12) = 11.1, *p* = 0.002) and “F2 on same or different electrode to F1” (F(1,6) = 29.7, p = 0.002) and, importantly, a highly significant interaction (F(2,12) = 24,2, *p* = 0.001). (These analyses excluded the data point with F2 presented on e20, which was identical for the “same-electrode” and “different-electrode” conditions, and used the Huynh-Feldt sphericity correction). Figure 8 B shows that the same general trends occurred for all ears tested, although the degree of tuning differed across participants. Specifically, the NDR at a separation of 6 electrodes was smaller than that for same-electrode stimulation for three subjects (C09, C18, C27) but not for C19L, C19R, or C30.

### EASSR

**Fig. 9:**
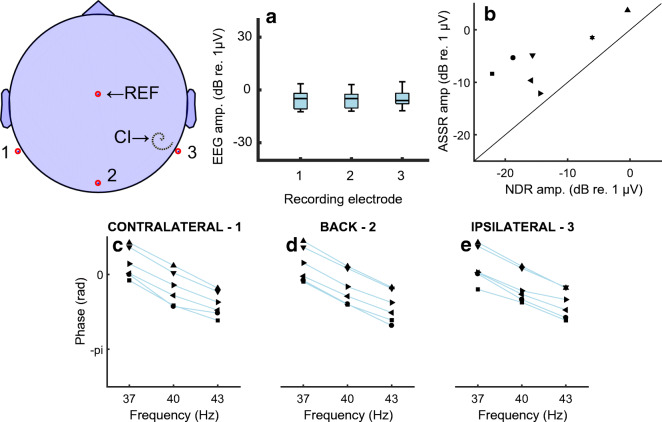
Parts A–D show the amplitude and phase-frequency plots for the EASSR measured from three different recording electrodes, shown schematically on the top left. The format of these panels and the naming of electrodes is the same as for the NDR plots in Fig. 7. Part E) shows a scatterplot of EASSR vs NDR amplitudes for eight ears tested

The EASSR for 500-pps amplitude-modulated pulse trains was measured using the same stimulating electrode and recording electrodes for seven of the ears from which we obtained the NDRs. For the EASSRs, unlike the ALFIES measures, recordings were processed so as to identify and blank the stimulus pulses in the recording and to linearly interpolate across these blanked values (Gransier et al. 2020). In contrast to the findings of Gransier et al. (2016), obtained using similar stimuli, we were able to observe an EASSR that was dominated by a neural response, rather than by electrical artefact, even from recording electrodes ipsilateral to the implant. Both the EASSR amplitude (Fig. 9A) and the function relating EASSR phase to modulation frequency (Figs. 9C–E) were similar for the ipsilateral, contralateral, and back recording electrodes. The average group delay was 44 ms, very close to the 45 ms observed for the NDR. We attribute our ability to measure an EASSR from all electrodes to our use of a hyper-rate EEG system, which allows better sampling of the electrical artefact associated with each pulse. Note, though, that when using the same recording system but with a higher pulse rate of 900 pps, we could only measure the EASSR from recording electrodes contralateral to the CI (Gransier et al. 2020). Hence, the ability to record an EASSR (but not an NDR using ALFIES) appears to depend on a combination of the amount of stimulus current reaching the recording electrode, the pulse rate, and on the accuracy with which one can sample the artefact. Figure 9 B shows that the 500-pps EASSRs were consistently larger than the 4644-pps NDRs, and the two measures were significantly correlated across ears (*r* = 0.79, df = 5, *p* = 0.03). The difference in the amplitudes of the two measures, averaged across all ears tested, was 7.9 dB (*t*(6) = 4.45, *p* < 0.01).

## Discussion

The ALFIES method described here provides a fast measure of a sustained neural response that can be obtained using the highest pulse rates employed clinically and using a single pair of recording electrodes, even for a recording electrode adjacent to the CI. It reveals a neural nonlinearity that is unobscured by the nonlinear response of the basilar membrane and permits one to measure the spatial selectivity of the sustained response. This section compares the ALFIES measures to the EASSR and then discusses the source of the neural nonlinearity that we observe. It then relates our results to other measures of spatial selectivity in CI users, discusses potential sources of artefactual distortion products, and considers potential applications of the ALFIES method both for CIs and for other forms of electro-neural stimulation.

### Comparison with the EASSR

The only other measure of the sustained thalamic/cortical response to CI stimulation is the electrically evoked auditory steady-state response (EASSR). Hofmann and Wouters (2012) measured the EASSR using amplitude-modulated pulse trains that were presented in bipolar mode so as to limit current spread. Gransier et al. (2016) subsequently obtained EASSRs for amplitude-modulated 500-pps pulse trains, presented in monopolar mode, which is the mode of stimulation used clinically in all modern CIs. To remove the electrical artefact, they removed time periods corresponding to each electrical pulse from the recorded stimulus and linearly interpolated across these blanked points. We used the same method to measure the EASSR at AM rates of 37, 40, and 43 Hz in seven of our tested ears and obtained an average group delay of 44 ms, very similar to that observed for the NDRs of about 45 ms obtained with the ALFIES method. The EASSRs were larger than the NDRs (Fig. 9E) but a direct comparison between the size of the response obtained with the two measures is hindered by the different pulse rates used, and from the fact that it would not be possible to measure an EASSR at the 4644-pps pulse rate used for the ALFIES measures. Recently, by using the same hyper-rate BioSemi system employed here, Gransier et al. (2020) achieved more accurate sampling of the artefact so as to measure an EASSR to amplitude-modulated monopolar stimuli with a carrier rate of 900 pps. This will allow us in the future to compare the amplitudes of the NDR and the EASSR using similar carrier rates. Meanwhile, we note that the ALFIES method uniquely allows one to record from locations close to the CI at pulse rates at least as high as 4644 pps, whereas, even with a hyper-rate EEG system, the maximum rate for EASSR lies between 500 and 900 pps.

It is also worth remarking that, although we used a hyper-rate (> 200 kHz) recording system, this is not necessary for the ALFIES method. EEG systems usually apply a low-pass antialiasing filter prior to digitization, but the resulting smoothing, by itself, will not introduce a distortion product whose frequency depends on both F1 and F2. This is because, for interleaved stimuli, distortion products will only arise from nonlinearities that occur *after* smoothing. However, any nonlinearities in the recording system that occur after smoothing by the brain or by the antialiasing filter could produce a DP. This can be avoided by using any EEG system that is linear over a wide dynamic range; this includes the hyper-rate system used here but other linear systems will be sufficient and a very high sampling rate is not necessary. Of course, it is always prudent to check that the nonlinearity is of neural origin, using methods such as described here—namely measuring the group delay, examining the distribution of response amplitudes for different recording electrodes, and/or varying the overall frequency of stimulation.

### Sources of Neural Nonlinearities in the Auditory System

An additional feature of our results is that they reveal nonlinearities in the auditory system that are entirely of neural origin. Neural nonlinearities are likely to make a substantial contribution to the distortion components observed in phase-locked measures of the response to acoustic stimulation, but in that case, basilar membrane nonlinearities may also play a role (Krishnan 1999; Gockel et al. 2012). Neural nonlinearities can also be revealed by presenting an acoustic stimulus diotically and comparing the resulting EEG trace to the sum of the traces obtained with monaural presentation at each ear separately (Dobie and Norton 1980; McPherson and Starr 1993; Gransier et al. 2017). In that case, cochlear nonlinearities are present but are unaffected by the manipulation, and this “binaural interaction component” technique measures (only) those nonlinearities that occur at and after the site of binaural integration. In contrast, CI stimulation bypasses cochlear processing, allowing the ALFIES technique to reveal neural nonlinearities at all stages of the auditory system, unaffected by basilar membrane mechanics.

Although the NDR that we observed for F2-F1~40 Hz had a group delay consistent with a cortical and/or thalamic origin, we should stress that the smoothing and nonlinearity responsible for its production could have occurred at any stage of the auditory pathway up to and including auditory cortex. Responses generated at each stage of auditory processing, including the post-synaptic potentials responsible for cortically generated EEG potentials, will likely inherit the nonlinearities generated at earlier processing stages, and each stage may introduce further temporal dependencies and nonlinearities. It is worth noting that interleaved stimuli with F2-F1 Hz between 90 and 120 Hz would also have been subjected to smoothing followed by nonlinearity. The absence of an NDR at those rates may have been due to the brainstem generators responsible for EEG components at 90–120 Hz rates being far from the scalp, and/or because phase locking in the brainstem of CI listeners may not extend to higher rates (Gransier et al. 2016).

As mentioned in the Introduction, smoothing initially occurs as charge is integrated at the auditory nerve membrane. Psychophysical experiments have shown that, when two pulses are presented in close succession, the masking of one pulse by another depends on their relative polarity for inter-pulse delays up to several hundreds of microseconds, with a biphasic pulse being more easily detected when its first phase has the same polarity as the second phase of the preceding masker compared with when they have opposite phases (de Balthasar et al. 2003; Karg et al. 2013; Cosentino et al. 2015a; Macherey et al. 2017; Guérit et al. 2018). These interactions must occur at the auditory nerve membrane and can affect masking for inter-pulse gaps up to 1 ms, with the largest effects observed for gaps less than 240 μs (Cosentino et al. 2015a). The interleaved pulse trains used in our experiments had a composite pulse rate of 4644 pps and a pulse duration of 58 μs, leading to an inter-pulse gap of 157 μs. There would therefore have been sufficient smoothing, even at the level of the auditory nerve, for nonlinearities to produce an NDR for our interleaved pulse trains. This does not of course mean that subsequent smoothing, for example that arising from synaptic transmission at more central stages of the auditory system, did not play a role. Other temporal dependencies, such as refractoriness and/or adaptation, would also cause the neural representation of the stimulating waveform at each time point to depend on earlier parts of the waveform, which would have contained both the F1-Hz and the F2-Hz components. This would have made it possible for subsequent neural nonlinearities to produce a response at frequencies, such as F2-F1 Hz, that depend on both F1 and F2 Hz. Hence, although the initial smoothing and rectification involved in the initial transformation from electrical stimulation to auditory nerve action potential would have been sufficient, in principle, to produce an NDR, the size of that NDR may well depend on temporal dependencies and nonlinearities at multiple stages of the auditory system.

### Spatial Selectivity of CI Stimulation

Ideally, each channel of a CI would excite a discrete set of auditory neurons close to that electrode, and this pattern of spatial selectivity would be preserved throughout the auditory system. In practice, spatial selectivity is degraded by a number of factors, including current spread within the cochlear fluids, deterioration or death of auditory neurons, and long-term central changes that may arise from the period of auditory deprivation prior to implantation (Kral et al. 1998; Vollmer et al. 2007; Fallon et al. 2008; Goldwyn et al. 2010; Kalkman et al. 2015). This section compares the selectivity measure provided by the ALFIES method with existing psychophysical and electrophysiological measures of spatial selectivity.

#### Behavioural Measures of Spatial Selectivity

Most psychophysical experiments that have measured spatial selectivity have used a masking paradigm. The benefits and shortcomings of different masking methods have been discussed in detail elsewhere (McKay 2012; Cosentino et al. 2015a). Here we simply note that, in all masking experiments, successful detection requires the probe, when added to the masker, to produce an excitation pattern that differs from that produced by the masker alone. This means that such measures will be largely determined by the areas of the masker + probe and probe-alone excitation patterns that do *not* overlap (e.g. by the addition of the probe producing a local increment in the masker-alone excitation pattern). Similarly, tasks that require listeners to discriminate between sequential stimulation of two electrodes are dominated by the areas of the two excitation patterns that do not overlap. In contrast, the NDRs produced using the ALFIES method and with F1 and F2 presented to separate electrodes depend on the parts of the excitation patterns, elicited by stimulation of the two electrodes, that *do* overlap. As a consequence, when F1 and F2 are presented, say, 6 electrodes apart, the presence of an NDR requires only a 3-electrode-wide spread from each stimulating electrode. This may partly account for why the tuning curves in Fig. 8 appear quite broad for some listeners, who show substantial NDRs even for a six-electrode separation.

The behavioural measure of selectivity most similar to that provided by ALFIES comes from a paradigm that does, in principle, reflect the overlap between two excitation patterns (McKay and McDermott 1996, 1999; Macherey and Carlyon 2010; Fielden et al. 2013; Marozeau et al. 2015). The paradigm uses pairs of low-rate (e.g. 100 pps) pulse trains, presented to separate electrodes, and requires listeners to discriminate a standard stimulus, where the temporal offset between the two pulse trains is close to zero, from one where it has a longer value. When the electrode separation is small, listeners hear a difference in temporal pitch between the two stimuli, but performance becomes increasingly worse as the electrode separation increases. One difference between this measure and ALFIES is that the behavioural measure uses a pulse rate much lower than that used clinically. Another difference is that good performance depends not only on the existence of neurons that respond to the combined temporal patterns of the two electrodes, but also on this combined response substantially affecting pitch, despite the likely presence of other areas (e.g. apical to the more apical electrode, and basal to the more basal one) that convey only the simple pulse rate applied to one electrode.

#### Electrophysiological Measures of Spatial Selectivity

Here, we consider two other electrophysiological measures of spatial selectivity in CI listeners. The electrically evoked compound action potential (ECAP) is a composite measure of synchronized auditory nerve activity. It is often measured using the masker-probe method (Brown et al. 1990), in which the response to a probe (P) and to a masker (M) pulse is obtained both separately and with the masker immediately preceding the probe (MP). If the masker completely suppresses the response to the probe then the subtraction (M + P − MP) gives the neural response to the probe, whilst cancelling the artefact. If the masker has no effect on the probe response the subtraction yields a zero ECAP, and several authors have measured selectivity as the decrease in the ECAP as the separation between the masker and probe electrodes is increased (Cohen et al. 2003; Hughes and Stille 2010; Cosentino et al. 2015b; Biesheuvel et al. 2016; Spitzer et al. 2019). Because the ECAP depends both on the size of the response to the probe and to it being masked, it has been argued that this method measures the overlap in the masker and probe excitation patterns, as is the case when spatial selectivity is measured using ALFIES (Cosentino et al. 2015b; Biesheuvel et al. 2016; Garcia et al. submitted.). One difference between the ALFIES and ECAP measures is that, of course, ECAPs measure the response at the level of the auditory nerve whereas the NDRs shown in Fig. 8 were generated at the auditory cortex and/or thalamus. The second is that ECAPS are usually measured at pulse rates of 80 pps or slower and measure a transient response, whereas the ALFIES method measures a sustained response to high-rate pulse trains. This may be relevant to estimates of selectivity because of evidence from inferior colliculus recordings that the transient response may be less spatially selective than the sustained response (Schoenecker et al. 2012). For these reasons, ALFIES may provide a better correlate than ECAPs of the effects of spatial selectivity on auditory perception, especially with the pulse rates used clinically. An advantage of the ECAP method is that it is very fast and requires no extra equipment.

Another objective method, the Electrically evoked Auditory Change Complex (EACC) measures the thalamic and/or cortical evoked response to any change in an ongoing stimulus, including a change in the stimulating electrode (Brown et al. 2008; He et al. 2014; Mathew et al. 2016; Mathew et al. 2017). The EACC response is not specific to changes in place-of-excitation and can, for example, be elicited by a change in level. This makes it important, in studies of spatial selectivity, to carefully equate the loudness of the two electrodes to be stimulated sequentially; this is less important for ALFIES. In addition, as noted above, the transient response to the change in stimulating electrode may exhibit a different selectivity to the (arguably more perceptually relevant) sustained response. Finally, as is the case for behavioural measures of electrode discrimination, the EACC measures the response to portions of the two excitations that do not overlap; hence, ALFIES and the EACC measure different aspects of spatial selectivity.

### Practical Applications

The most likely practical application of the ALFIES method for CIs is that it can measure the selectivity of the sustained auditory response at the level of the auditory cortex and/or thalamus. CI patients differ substantially in their ability to perceive speech, probably due to a combination of sensory and cognitive factors (Zhao et al. 2020), and this ability can also be affected by the patient’s experience with speech prior to and following implantation. The clinician, faced with a patient having very poor speech understanding, has to disentangle these various factors in order to identify appropriate diagnoses and treatment. Preserved thalamic/cortical tuning, as revealed by the ALFIES method, combined with poor speech perception could therefore indicate a cognitive or language-based, rather than a sensory, basis for this poor performance.

ALFIES might also prove useful in evaluating novel interventions that can only initially be tested in animals, such as penetrating electrode arrays and pharmaceutical treatments (Middlebrooks and Snyder 2007; Wise et al. 2016; Plontke et al. 2017). Evaluation of such methods often records the brainstem transient response to single pulses, in a terminal experiment. In contrast, ALFIES provides a non-invasive measure of the selectivity of the sustained response at the thalamic/cortical level, using stimuli close to those that would eventually be used in clinical settings. The same measure can then be employed if and when the new method is transferred to humans. Its robustness to small distances between the recording electrode and the stimulating device makes it suitable for testing with animals, regardless of head size.

Finally, we note that the usefulness of the ALFIES method may extend beyond CIs and into non-auditory applications. Electrical stimulation of the brain and nervous system is used in fields as diverse as retinal implants, deep-brain stimulation for the alleviation of neurological disorders, transcranial alternating-current stimulation to investigate cognitive function, and intra-cortical stimulation of epilepsy patients for the characterization of language networks (Matsumoto et al. 2004; Walker et al. 2012; Zrenner 2013; Noury et al. 2016; Neuling et al. 2017). All of these applications use either biphasic pulse trains or sinusoids, whose parameters differ quantitatively but not qualitatively from those used here and illustrated in Figs. 1 and 2. In each case, there is a need to measure the neural response to that electrical stimulation, in the presence of substantial artefact, and in at least some applications, the ability of existing methods to do so remains contentious (Noury et al. 2016; Neuling et al. 2017). We are currently investigating whether the ALFIES method can be successfully applied to these non-auditory domains.

## Supplementary Information

ESM 1 (DOCX 22 kb)

## Footnotes

1 https://github.com/d-mao/trigger-box
