# Supplementary material for: Using Interleaved Stimulation to Measure the Size and Selectivity of the Sustained Phase-Locked Neural Response to Cochlear Implant Stimulation
Source: J Assoc Res Otolaryngol. 2021 Jan 25;22(2):141–59. doi: 10.1007/s10162-020-00783-y (PMC7943679; doi:10.1007/s10162-020-00783-y)
Supplement: Supplementary file 1 — (DOCX 22 kb) [file 10162_2020_783_MOESM1_ESM.docx]

**APPENDIX: TEXT OF CONSENT FORM SIGNED BY PARTICIPANTS**

**CONSENT FORM**

Title of Project: **Segregating concurrent sounds in acoustic and electric hearing**

Chief Investigator: Dr Robert Carlyon, University of Cambridge

IRAS Number: 118230

Please initial box

1. I confirm that I have read the information sheet dated......................... for the

above study. I have had the opportunity to consider the information, ask questions and have had these answered satisfactorily.

1. I understand that my participation is voluntary and that I am free to withdraw at any time without giving any reason, without my medical care or legal rights being affected.
2. I understand that sections of my medical notes and data collected during
   the study may be viewed by individuals from the University of Cambridge, where it is relevant to my taking part in this research. I give permission for these individuals to have access to my records.
3. I understand that the information collected about me will be used to support
   other research in the future, and may be shared anonymously with other researchers.
4. I agree to take part in the above study.

Name of Participant Date Signature

Name of Person Date Signature

taking consent
